# Stress Concentration Factors for Butt-Welded Plates Subjected to Tensile, Bending and Shearing Loads

**DOI:** 10.3390/ma13081798

**Published:** 2020-04-11

**Authors:** Krzysztof L. Molski, Piotr Tarasiuk

**Affiliations:** Faculty of Mechanical Engineering, Bialystok University of Technology, Wiejska 45C, 15-351 Bialystok, Poland; k.molski@pb.edu.pl

**Keywords:** butt-welded joints, notch, stress concentration factor, weld toe, finite element analysis, axial, bending and shearing load

## Abstract

This paper deals with the analysis of stress concentration at the weld toe of a Double-V and a Single-V butt-welded joints subjected to tensile, bending and shearing loads. For each geometrical and loading case accurate close form stress concentration factor formula based on more than 3.3 thousand finite element method solutions were obtained. The percentage error of the formulas is lower than 2.5% for a wide range of values of geometrical parameters including weld toe radius, weld width, plate thickness and weld toe angle. The limiting case, in which the weld toe radius tends to zero is also considered. In the cases of shearing loads, a plane model based on thermal analogy was developed. The whole analysis was performed assuming that a circular arc represents the shape of the excess weld metal. Presented solutions may be used in computer aided fatigue assessment of structural elements.

## 1. Introduction

Welded joints are one the most commonly used types of connection. Years of development in production technology have increased such qualities as—improved tightness, low cost and shorter fabrication times. However, some unfavorable phenomena can still be observed at the weld zone, such as structural irregularities and imperfections of the material, residual stresses, cracks or undercut defects that may cause a significant decrease in the fatigue life of the structure. Many scientific works that relate to physical fatigue phenomena, modelling of damage processes and durability calculations have dealt with the topic of strength of welded joints and the development of appropriate design procedures.

Fatigue crack growth is a basic phenomenon occurring in welded structures subjected to variable loading where the fatigue propagation rate may depend on the crack length, weld geometry and accompanying residual stress field [1,2,3,4,5]. In such cases the fracture mechanics approach based on the stress intensity factor concept proves to be very convenient. An experimental method useful for determining the stress intensity factors for real welded structures has been presented by Chung et al. [6].

Numerous methods of fatigue analysis are based on local stress approach [7,8] and various concepts, such as—structural stress [7,9,10,11,12] and corresponding hot-spot stress [11,13,14,15], effective notch stress [10,16,17] related to the reference notch radii [10,12,18] and many others. Moreover, local plastic zone may be produced by a high stress concentration. In such cases cyclic plastic zone may serve as a proper parameter to determine more accurate predictions of fatigue life [19]. Influence of residual stresses on fatigue initiation period based on local approach was described in References [3,20,21]. Livieri and Lazzarin Reference [22] proposed a method for the assessment of fatigue strength of welded joints based on generalized tress intensity factors applicable to sharp v-notches. General problems related with notches can be also found in recently published review [23] on advances on notch effects in metal fatigue.

More complex models considering two stage damage phenomenon including fatigue crack initiation and propagation periods were presented in References [10] and [24].

Two approaches based on the nominal stresses and the notch stresses were studied in Reference [20] indicating the local approach being more appropriate for predicting fatigue life and fatigue strength.

Numerous standards and recommendations have also been developed to facilitate design [25,26,27,28,29,30].

One of the basic problems when trying to estimate the fatigue life of a structure is determining the maximum stress in order to transform the loading history of remote stresses to the weakest point, where fatigue cracking may be initiated. To this end, stress concentration factors (*SCF*s) (whose values depend on geometry and loading conditions) are commonly used [31]. Some research [32,33,34,35] has also provided analytical solutions for bodies of various geometries, subjected to different loading conditions.

The same problem of stress concentration holds for welded structures, where the weakest point is usually located in the weld zone. Therefore, many *SCF* solutions have been developed and published regarding various types of welded joints. Numerous formulas of stress concentration factors widely used in Japan for various types of welded joints were presented in References [36,37,38]. These approximating formulas were based mainly on numerical results obtained using the finite element FE and the boundary element BE methods. Extended numerical analysis for T-joints and skewed T-joints was performed by Brennan et al. [39] where two parametric equations were proposed. However, the authors defined SCFs in a different way considering the maximum stress at the transition point between the circular arc and the plate surface. Such a definition makes the SCFs values underestimated of about 7–9% with respect to the ones defined for the maximum principal stress at the curvilinear surface of the weld toe.

Additional effects of fabrication tolerances, misalignments, undercuts and so forth, are often included in the analysis. Theoretical values of SCFs for pipelines and pressure tanks, including fabrication tolerances, have been published by Lotsberg in References [40] and [41]. Effects of stress concentration in grinded regions of T-butt welded connections were presented in Reference [42]. Extended numerical finite element method (FEM) analysis of geometrical parameters and their impact on SCF in butt welded joints was shown in References [43,44,45]. Effects of misalignments in butt welded joints were discussed in References [46,47,48] and an analysis of undercut defect and reinforcement metal has been published in Reference [49]. Assessment of fatigue life requires high accuracy of *SCF* solutions because errors made when estimating maximum stresses (of just a few percent) can lead to tens or even several hundred percent inaccuracy in estimating fatigue life. For this reason *SCF* approximating formulas should be highly accurate and cover a wide range of values of all the basic parameters that influence *SCF*s.

The use of known approximation formulas in fatigue design encounters some difficulties arising from their accuracy, range of validity and different ways of defining *SCF*s, therefore, they should be used with appropriate caution.

The present work deals with the determination of stress concentration factors in the weld toe region of Double-V and Single-V, butt-welded joints that have been subjected to tensile, bending and shearing loads. An extended review of the published formulas dealing with SCFs for weldments subjected to tension and bending are presented in Reference [45] and they will not, therefore, be quoted here. The finite element method (*FEM)* modelling was used in the present work, with particular attention being paid to the accuracy and wide ranging validity of the developed approximation formulas, taking into account the limit case when the weld toe radius *ρ* tends to zero.

## 2. General Assumptions

**Figure 1:**
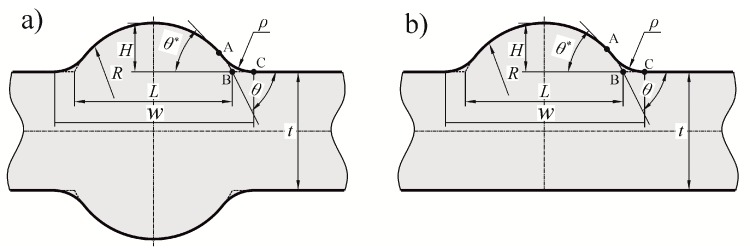
Shape and basic parameters of the butt welded joints: (**a**) a Double-V and (**b**) a Single-V.

Two types of butt-welded joints were analyzed—Double-V and Single-V. Each of them was subjected to tension/compression, bending and shear. The shapes and basic geometrical parameters of the full penetration butt-welded joints are illustrated in Figure 1.

The following assumptions have been made for all of the analyzed joints:

1. Joint material is linear elastic, isotropic and homogeneous
2. Small deformations occur due to external loading
3. Joint material is free from residual stresses, structural irregularities and imperfections
4. Both plates are of the same thickness *t* and are co-linear
5. Convex excess weld metal has a constant curvature, described by the radius *R*
6. The weld is symmetrical (for Single-V joint) or double-symmetrical (for Double-V joint)
7. Contour of the weldment is smooth, with a transition radius *ρ* > 0
8. Weld toe curvature and the excess weld metal curvature join at the point A (Figure 1)
9. SCF for tensile and bending loads is defined as *σ*_1max_/*σ*_t_ and *σ*_1max_/*σ*_b_, respectively
10. SCF for shearing load is defined as *τ*_max_/*τ*_s._

## 3. Numerical FEM Modelling and Some Numerical Results

### 3.1. Tensile and Bending Loading

**Figure 2:**
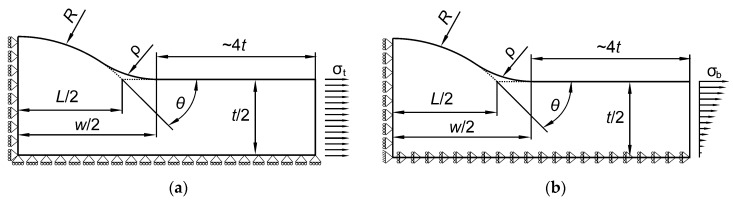
Boundary conditions for a Double–V butt-welded joint subjected to (**a**) tensile and (**b**) bending loads.

**Figure 3:**
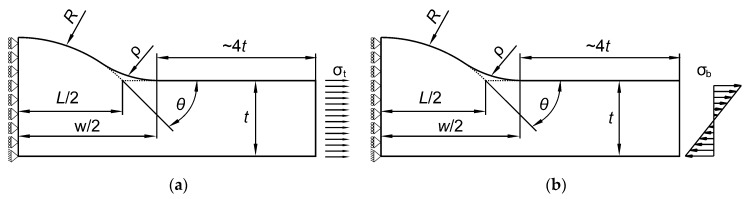
Boundary conditions for a Single–V butt-welded joint subjected to (**a**) tensile and (**b**) bending loads.

The ANSYS 19 *Multiphysics* program and PLANE182 finite element were used. PLANE182 finite element is defined by 4 nodes having 2 degrees of freedom at each node. The shape, loading and displacement boundary conditions for each type of welded butt joint are shown in Figure 2 and Figure 3.

The length of the modeled body is a vital parameter. According to the theory of elasticity and de Saint-Venant principle, if the distance of the load applied is sufficiently far from the weld toe (a few times larger than the element thickness in this case) there will be no differences in the values of the maximum stress. The proper length was found by conducting preliminary tests and finally the distance from the weld toe to the region of the load application was at least 4 times the thickness t.

**Figure 4:**
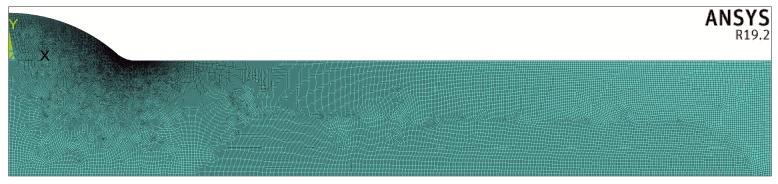
Example of a finite element mesh for X = 0.1, Y = 2/3 and θ = π/4.

**Figure 5:**
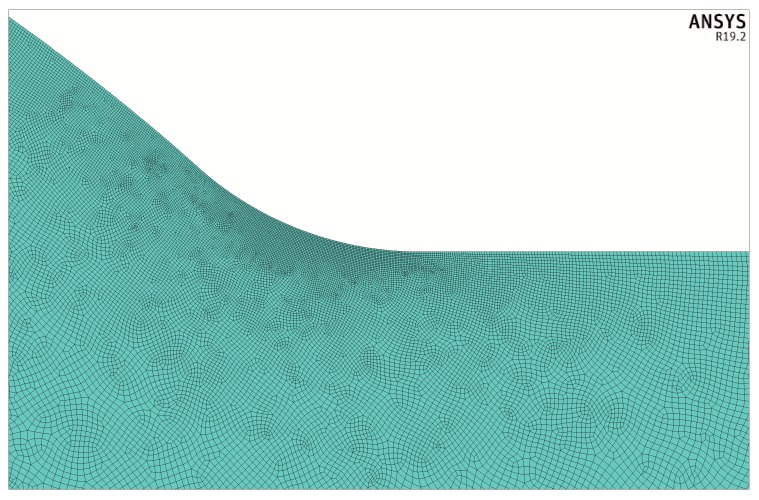
Finite element mesh in the weld toe zone for the case shown in Figure 4.

Approximately 900,000 finite elements were used for each model and special attention was given to the finite element mesh density at the weld toe zone. One example of such a mesh is shown in Figure 4 and Figure 5.

At the initial stage of building the FEM model the finite element mesh density was successively increased to obtain a stable numerical solution with the maximum stress value staying constant. As a result of gathering a significant number of cases and corresponding numerical solutions a special mesh generating procedure was developed. The number of elements along the notch radius was at least 100. Moreover, the size of the finite elements changed smoothly the further from the zone of maximum stress concentration. These assumptions made the finite element mesh very fine.

Number of finite elements, nodes and the minimum size of the element depended on the proportions between geometrical parameters of the joint represented by *X*, *Y* and *θ*. For a given geometry of the model, mechanical or thermal, the EF mesh was identical. For example, the model show in Figure 4, where *X* = 0.1, *Y* = 2/3 and *θ* = π/4, contained 1,076,779 elements and 1,078,547 nodes. The minimum size of the element with respect to the thickness t was 2.497 × 10^−5^.

Since the *SCF* values are the same and all dimensions of the body are proportionally changed, two non-dimensional parameters (*X* and *Y*) were introduced:

*X* = *ρ*/(*ρ* + *L*), (1)

*Y* = *L*/(*L* + *t*). (2)

Theoretical width *L* is a hypothetical distance between two symmetrical points, the intersection of the circular convex arc and the plate surface (Figure 1), while *θ* represents the theoretical weld toe angle at the same point, B. Extended numerical calculations were subsequently carried out in the following ranges—0.05 ≤ *X* ≤ 0.7, 0.075 ≤ *Y* ≤ 0.7, with intervals of 0.05 and, for 10° ≤ *θ* ≤ 90°, changing by 5°. More than 3300 *SCF* numerical results were obtained for each type of joint and loading mode. One example of such *SCF* results for a Single-V butt-welded joint, subjected to tension and for *θ* = 45°, is presented in Table 1.

### 3.2. Shearing Load

In the literature dealing with the strength of welded structures, only *SCF* solutions for tensile and bending loads are commonly available. There are two probable reasons for omitting such solutions for shearing loads. The first is that maximum local stress and corresponding fatigue crack initiation processes usually appear in the weld toe region, where the nominal stress is normal to the weld line and shear stress components may vanish. The second reason is that numerical *FEM* calculations of *SCF* for anti-plane problems are more difficult to carry out than in previous cases, due to the lack of 2D modules for solving such problems in commercial *FE* programs. However, in many practical cases, shear stress may make a significant contribution to the maximum effective stresses in the toe region. This may occur when external variable loads produce cyclic, non-proportional multi-axial stress states of varying principal directions or when the butt-weld is slanted with respect to the main load of the structure. In such cases, *SCF* for shearing load should also be considered in fatigue analysis. For these reasons, *SCF* solutions for shear have been taken into account in the present work.

The formulation of two-dimensional problems for anti-plane states of deformation is different than for in-plane tensile and bending loads. It is well known that any anti-plane case may be treated as a boundary value problem governed by the Laplace equation, represented in Cartesian coordinates as

(3) $$\frac{\partial^{2}\Psi }{\partial x^{2}}+\frac{\partial^{2}\Psi }{\partial y^{2}}=0,$$

where the potential function *Ψ*(*x*,*y*) is equivalent to the out-of-plane displacement function *W*(*x*,*y*). The fact that the same relationship also holds for the temperature field *T*(*x*,*y*), in plane thermal problems of steady-state heat flow, leads to the conclusion that *thermal analogy* may be used to obtain solutions of stress concentration factors for anti–plane shear. Thermal modules are widely accessible in the finite element commercial programs, making the modelling procedure simple and effective. Basic equations and material constants representing the analogy between the anti-plane shear and the steady-state heat conduction problem are shown in Table 2.

**Figure 6:**
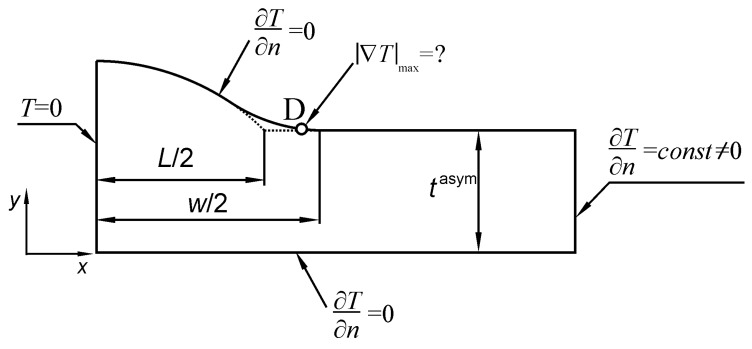
Boundary conditions for calculating stress concentration factors (SCFs) of a butt-welded joint subjected to anti-plane shear using thermal analogy.

The ANSYS 19 *Multiphysics* program with *Thermal* option and PLANE55 finite element were used in the present analysis. PLANE55 finite element is defined by 4 nodes with a single degree of freedom corresponding to temperature at each node. Meshing of the modelled area was the same as in the previous cases for tension and bending. The shape of the Single-V butt-welded joint model, as well as mixed boundary conditions, are shown in Figure 6. Nominal uniform heat flux q_nom_ was applied to the right end of the body, while zero temperature was applied to the left end. Since the upper face and the lower face of the joint are free from external shearing loads, they have to be insulated in the thermal model. The potential function *Ψ*(*x*,*y*), corresponding to anti-plane displacements *W*(*x*,*y*), is now represented by the temperature field *T*(*x*,*y*). It is clear that the shear stress components, related to the partial derivatives of the potential in particular directions, are proportional to the corresponding heat flux components.

Numerical *SCF* values are easily calculated as a ratio of the maximum magnitude of the temperature gradient |∇*T*|_max_ at the weld toe zone (point D in Figure 6) to the magnitude of the nominal temperature gradient |∇*T*|_nom_ over the right end of the body. It is well known that *SCF* values in such cases do not depend on the conductivity of the medium, therefore the same result is obtained by comparing corresponding heat flux quantities q_max_/q_nom_.

**Figure 7:**
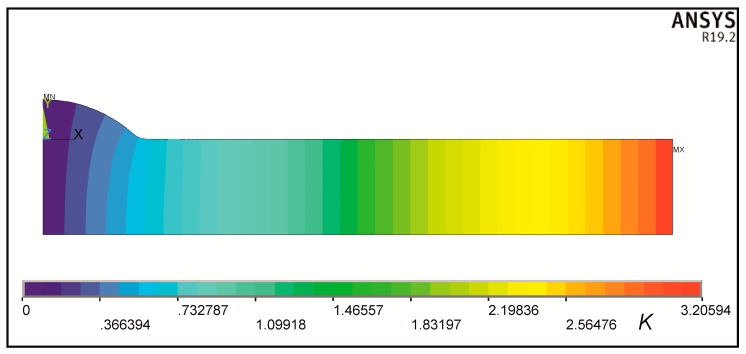
Temperature field and equipotential lines in heat conducting element.

**Figure 8:**
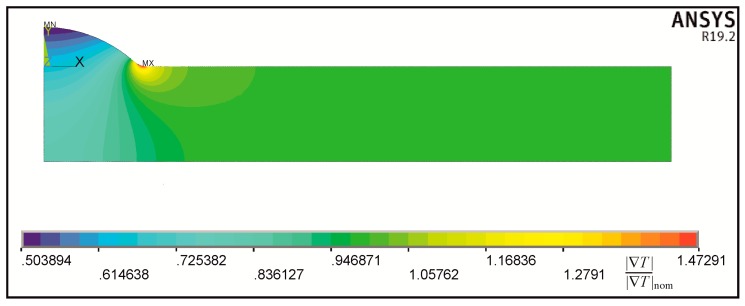
Magnitudes of the temperature gradient normalized with respect to |∇*T*|_nom_ = 1 and corresponding to the temperature field shown in Figure 7.

One example of a steady-state heat conduction solution is shown in Figure 7 and Figure 8. Temperature field and its equipotential lines are shown in Figure 7, where temperature values, interpreted as anti-plane displacements *W* of the body, increase from left to right. All equipotential lines are normal to both upper and lower faces.

Magnitudes of the normalized temperature gradient |∇*T*|/|∇*T*|_nom_ for |∇*T*|_nom_ = 1 and corresponding isolines are shown in Figure 8. This solution was obtained directly from ANSYS by recalling “thermal gradient vector sum” and can be interpreted as shearing stress *τ* resulting from anti-plane loading.

Qualitatively identical results can be reached for the heat flux magnitudes q, which also can be obtained directly by recalling “thermal flux vector sum.” Such solutions served in each geometrical case for calculating SCFs values.

**Figure 9:**
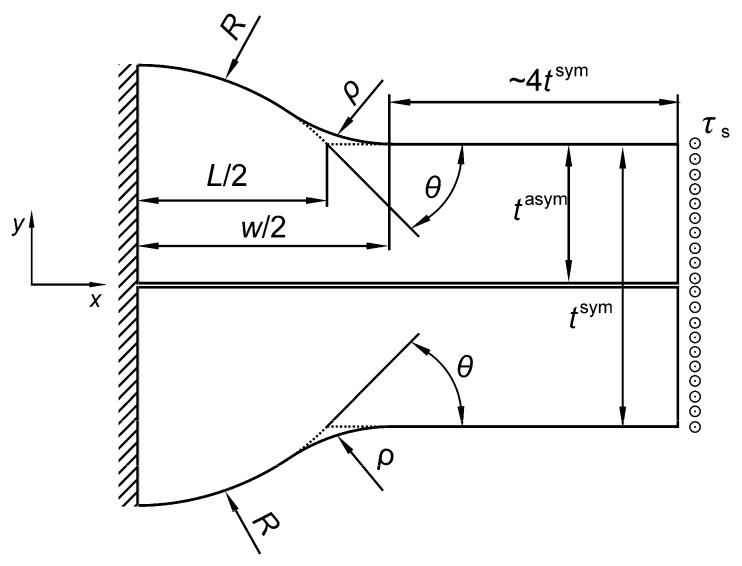
Boundary conditions for a Single–V and Double-V, butt-welded joint subjected to shear.

Such a formulation of the anti-plane problem has some additional consequences. Since the heat flux normal to the lower surface of the Single-V butt-welded joint equals zero (Figure 6), this surface may be considered as a plane of symmetry for a Double-V butt-welded joint, where displacement field *W*(*x*,*y*) is an even function about the *x* axis, as shown in Figure 9.

Hence, the two solutions of the *SCF* values for a Double-V and a Single-V butt-welded joint are identical, regardless of whether both halves of the body are joined together or separated, as shown in Figure 9. The only difference to consider when describing *SCF* values using approximating formulas, lies in defining the plate thickness *t*, indicated here as *t*^sym^ and *t*^asym^. By considering *t*^sym^ = 2*t*^asym^ and taking into account Equation (2), the relationship between *Y*^sym^ and *Y*^asym^ is

(4) $$Y^{sym}=\frac{Y^{asym}}{2-Y^{asym}\;}.$$

## 4. *SCF* Approximating Formulas

### 4.1. Singularity Effects at the Weld Toe

**Figure 10:**
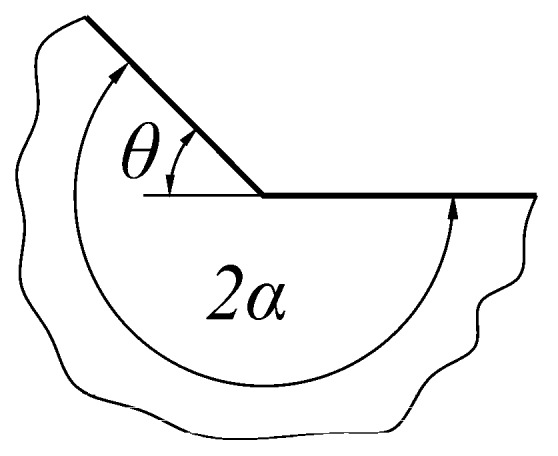
Characteristic angles 2*α* and *θ* of the sharp corner.

Correct description of the limiting case, when the weld toe radius *ρ* tends to zero, is a necessary condition for obtaining parametric functions when approximating *SCF* values for a given weldment geometry and loading conditions. In such a case, the weld toe region is transforming into a sharp corner, as shown in Figure 10, producing theoretically infinite stress at the apex. Thus, the main objective now is to find the correct relationship between the radius *ρ* and the maximum principal stress *σ*_1max_ at the notch root.

In cases of tensile or bending loading, the characteristic equation for *λ* [50,51] is given by Equation (5):

(5) sin(2αλ)+λsin(2α)=0.

while, for anti-plane shear, the characteristic equation [51] is:

(6) $$\cos\left(\lambda_{s}\alpha \right)=0.$$

Two characteristic quantities, *λ* and *λ*_s_, represent exponents of the displacement fields for normal and shear loads, respectively. This leads to the conclusion that every approximating function should contain an exponential term of the form *X*^n^, corresponding to the strength of the singularity in the limiting case, when the toe radius *ρ* tends to zero. Since 2*α* = π + *θ* (as shown in Figure 10), the *n* value should depend on the *θ* angle related to the eigenvalues, *λ* and *λ*_s_, obtained from Equations (5) and (6). It is also clear that the quantity *σ*_1max_*ρ*^n^ should have the unit MPa (mm)^1−λ^ which corresponds to that of the generalized stress intensity factor of the sharp corner [51]. Thus, particular values of both exponents, *n* and *n_s_*, may be obtained from Equations (7) and (8):

(7) n=λ−1

(8) $$n_{s}=\lambda_{s}-1.$$

**Figure 11:**
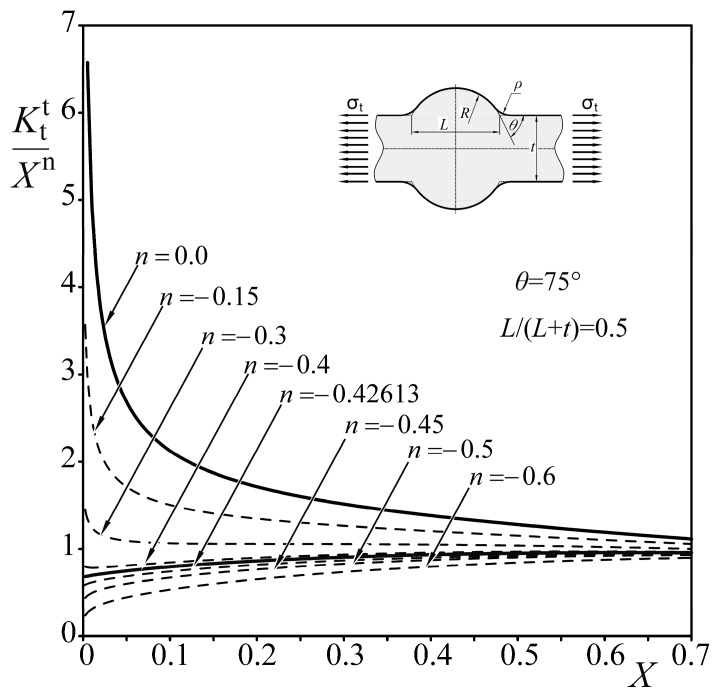
Influence of exponent *n* on the normalized *K*_t_^t^ of a Double-V butt-welded joint under tension, when *Y* = 0.5 and *θ* = 75°.

**Figure 12:**
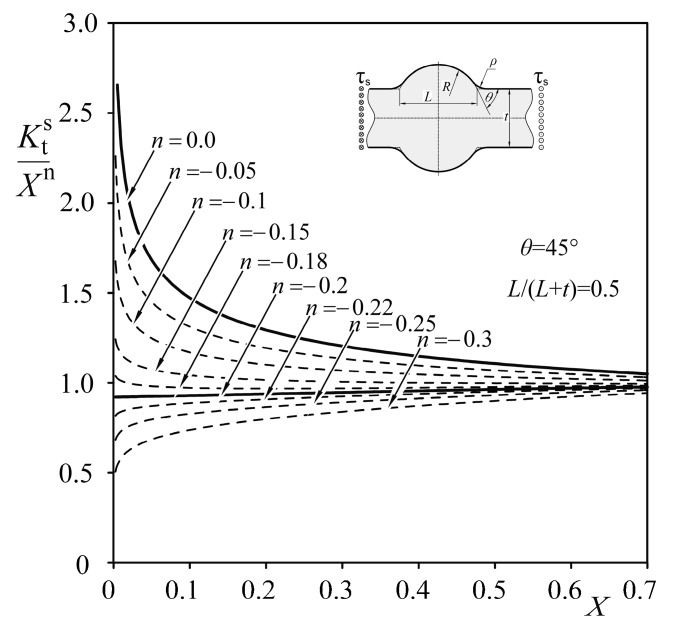
Influence of exponent *n* on the normalized *K*_t_^s^ of a Double-V butt-welded joint subjected to shear, when *Y* = 0.5 and *θ* = 45°.

The correctness of such a conclusion is presented in Figure 11 and Figure 12, where particular *SCF* values (for *n* = 0) obtained using *FEM* for a Double-V butt-welded joint subjected to tension and shear, respectively, are normalized by the term *X*^n^ for arbitrarily chosen *n* values. Only the exponents, *n* = −0.42613 and *n*_s_ = −0.2, corresponding to theoretical solutions, lead to the finite limits at *X* = 0. For higher exponents than these theoretical ones, normalized stress concentration factors, *K*_t_/*X*^n^, are infinite; for lower exponents, they equal zero.

Unfortunately, an analytical solution of the characteristic Equation (5) is not known and approximating numerical procedures need to be applied for calculating *λ*. Thus, Equation (9), based on numerical solutions of Equations (5) and (7), is proposed:

Equation (9) is valid in the range 0 ≤ *θ* ≤ π/2, representing values of exponent *n* with an accuracy of 5 significant digits.

(9) $$n=\frac{-0.63662\theta -0.0933\theta^{2}}{1+0.77635\theta +0.04075\theta^{1.5}-0.00499\theta^{2}+0.13365\theta^{2.5}}.$$

For shearing load, the exact solution of Equations (6) and (8) gives

(10) $$n_{s}=\left(\frac{-\theta }{\pi +\theta }\right).$$

where *θ* is in radians.

Results of the analysis presented above have proven that particular values of *n* and *n_s_* depend on the angle *θ* and the loading mode. The application of constant exponents in approximating formulas for *SCF* is not correct and leads to inaccurate results for relatively small weld toe radii.

### 4.2. General Form of the SCF Formulas

After normalizing the numerical results, with respect to the singular term *X*^n^ (as shown in Figure 11 and Figure 12), the *SCF* values for any particular joint may be represented by a regular function *P*(*X*,*Y*,*θ*), depending on the geometry and loading mode. Thus, the general form of the *SCF* approximating function is:

(11) $$K_{t}=X^{n}P(X,Y,\theta ).$$

Six particular *SCF* formulas for Double-V and Single-V butt-welded joints, each subjected to tensile, bending and shearing loads, are presented in the Appendix A. For each case of the function *P*(*X*,*Y*,*θ*), the number of terms and values of the exponents were chosen using a “step by step” approach in order to find the best qualitative representation of the known, normalized numerical *K*_t_/*X*^n^ values with respect to *X*. In the next step, all coefficients for particular terms were obtained by means of the least squares method. The procedure was then successively repeated for the other variables, *Y* and *θ*. In spite of the fact that the approximating procedure of *P* functions is sometimes troublesome and time consuming, it allows us to control accuracy and minimize the number of terms.

### 4.3. Validation of Approximating Formulas

Numerical *FEM SCF* values have been compared to their equivalencies obtained by means of the approximating functions. Some examples of such comparisons are presented in Table 3, Table 4 and Table 5 for a Double-V butt welded joint of *θ* = 30°, subjected to tensile, bending and shearing loads.

Accuracy for all approximating formulas and variables in the range of validity shown in the Appendix A, is better than 97.5%.

## 5. Discussion

### 5.1. Transformation of the Measurable Weld Parameters into Theoretical Ones

Previously defined variables *X*, *Y* and *θ*, are very suitable for theoretical analysis but from the point of view of engineering applications, some measurable parameters (as identified in real structures) are necessary. *L* and *θ* are inaccessible from the weld surface and it is much better to work with the total width w and the weld toe angle θ* defined in Figure 1. In spite of the fact that the values of both angles *θ* and θ* (as well as lengths *L* and *w*) are almost the same for small weld toe radii, they formally represent different quantities and should be treated separately.

Unfortunately, the assumptions of constant curvature of the excess metal and smoothness of the weld contour make the geometrical quantities of the chosen weldment mutually related. For instance, parameters w and θ* are enough to determine *H*. The same holds for *R*, *ρ* and *w*.

(12) $$H=\frac{w}{2}Tan\left(\theta^{*}/2\right)=R+\rho +\sqrt{{\left(R+\rho \right)}^{2}-\frac{w^{2}}{4}}.$$

This means that smaller or higher *H* values are not freely accessible, as long as all of the geometrical conditions defined in Figure 1 are satisfied. However, the constant curvature described by the *R* radius is close to the real shape of the weld and should give a good approximation of the real shape.

Other formulas, expressing various relations between geometrical parameters of the butt-welded joint, are as follows:

(13) $$\frac{R}{H}=\frac{1}{8}{\left(\frac{w}{H}\right)}^{2}-\frac{\rho }{H}+\frac{1}{2}$$

(14) $$L=H\sqrt{{\left(\frac{w}{H}\right)}^{2}-8\frac{\rho }{H}}=\sqrt{w^{2}-8H\rho }$$

(15) $$\theta =arcsin\left(\frac{\sqrt{{\left(\frac{w}{H}\right)}^{2}-8\frac{\rho }{H}}}{\frac{1}{4}{\left(\frac{w}{H}\right)}^{2}-2\frac{\rho }{H}+1}\right).$$

Equations (12) to (15) make it possible to transform the measurable parameters into the theoretical ones and calculate *SCF*s using the sets of formulas given in the Appendix A.

### 5.2. Comparison of the Present SCF Results with Other Solutions

**Figure 13:**
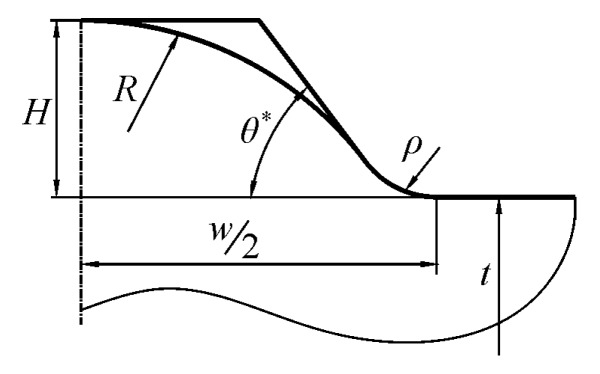
Comparison of two shapes of a butt-welded joint with the same *H*, *w*, *ρ* and θ*.

Parametric equations for calculating *SCF*s for a Single-V and a Double-V butt-welded joint, subjected to tensile and bending loads, have been published in Reference [45]. The authors analyzed the height of the excess weld metal and its influence on *SCF* values, assuming that the upper part of the weld is plane and *w*/*t* = 1.46. Such a weld shape is shown in Figure 13, compared to the circular one of radius *R* considered in the present analysis. All remaining geometrical parameters (*H*, *w*, *ρ* and θ*) are the same.

For the plane weld, the following parametric equation has been proposed [45]:

(16) $$K_{t}^{t}=1+1.3905{\left(\frac{H}{t}\right)}^{0.2081\theta^{*}}{\left(\theta^{*}\right)}^{1.0756}EXP\left[-1.7483\theta^{*}\right]{\left(\frac{\rho }{t}\right)}^{-0.259\theta^{*}}{\left(0.021+\frac{\rho }{t}\right)}^{-0.4413}$$

which results in *SCF* values with 96% accuracy.

A comparison of two *SCF* solutions given by Equations (16) and (A4) for a Single-V butt-welded joint under tension (for *w*/*t* = 1.46), is shown in Table 6. Corresponding *SCF* values are almost the same. More noticeable differences only appear for relatively small toe root radii (*ρ*/*t* < 0.05 for θ* = 60°).

Similar results were obtained for bending loads on a Single-V, butt-welded joint and for tensile and bending loads of a Double-V butt-welded joint, making use of other formulas in the Appendix A and those presented in Reference [45], corresponding to each case. This leads to the conclusion that both solutions are correctly performed and that the shape of the upper part of the excess weld metal has no significant influence on *SCF*s, if all remaining geometrical parameters of the joint are the same.

## 6. Conclusions

After performing extended numerical *FEM* modelling including about 20,000 cases, six approximating formulas for *SCF*’s covering both geometrical types of butt-welded joints and three independent loading modes were derived. The accuracy of the formulas is better than 97.5%, while the ranges of applications for the toe radius *ρ*, weld width *L*, plate thickness *t* and weld toe angle θ, are: 0 < *ρ*/*L* ≤ 2, 0 ≤ *L*/*t* ≤ 2 and 0 ≤ *θ* ≤ π/2 covering all geometrical situations occurring in engineering applications including the limiting case, when the weld toe radius *ρ* tends to zero.

Calculated *SCF*’s values agree very well with those obtained from parametric equations derived by Kiyak et al. in Reference [45] for some particular shapes of butt-welded joints.

In the cases of shearing loads, a plane *FEM* model developed for anti-plane problems and based on thermal analogy appeared to be very effective.

All the formulas derived here may be easily used in computer aided design for fatigue assessment of butt-welded joints.

## Figures and Tables

**Table 1:** Representation of *SCF* values for a Single-V butt joint subjected to tensile loading.

*θ* = 45°	*X* = *ρ*/(*ρ* + *L*)													
*Y* = *L*/(*L* + *t*)	0.05	0.075	0.10	0.15	0.20	0.25	0.30	0.35	0.40	0.45	0.50	0.55	0.60	0.65
0.075	2.155	2.223	2.041	1.816	1.676	1.578	1.505	1.446	1.397	1.355	1.319	1.287	1.257	1.230
0.10	2.515	2.221	2.040	1.815	1.676	1.578	1.504	1.445	1.396	1.355	1.318	1.286	1.256	1.229
0.15	2.509	2.217	2.036	1.812	1.672	1.575	1.501	1.442	1.393	1.351	1.315	1.282	1.253	1.225
0.20	2.502	2.210	2.029	1.805	1.667	1.569	1.495	1.436	1.387	1.345	1.309	1.276	1.246	1.218
0.25	2.488	2.198	2.017	1.795	1.657	1.559	1.486	1.427	1.378	1.336	1.299	1.266	1.236	1.207
0.30	2.466	2.179	2.000	1.779	1.642	1.545	1.472	1.413	1.364	1.322	1.285	1.252	1.222	1.193
0.35	2.436	2.152	1.976	1.757	1.621	1.526	1.453	1.394	1.346	1.304	1.267	1.234	1.204	1.175
0.40	2.395	2.116	1.942	1.728	1.594	1.500	1.428	1.370	1.322	1.281	1.244	1.212	1.182	1.154
0.45	2.340	2.068	1.898	1.688	1.558	1.466	1.396	1.340	1.293	1.253	1.218	1.186	1.158	1.132
0.50	2.269	2.006	1.842	1.640	1.514	1.425	1.358	1.304	1.259	1.222	1.189	1.159	1.133	1.110
0.55	2.182	1.931	1.774	1.582	1.463	1.379	1.316	1.265	1.224	1.189	1.159	1.133	1.110	1.090
0.60	2.081	1.844	1.697	1.517	1.406	1.329	1.271	1.226	1.189	1.158	1.132	1.110	1.090	1.073
0.65	1.969	1.749	1.614	1.449	1.348	1.279	1.228	1.188	1.156	1.130	1.108	1.089	1.073	1.060
0.70	1.852	1.651	1.529	1.381	1.292	1.231	1.187	1.153	1.126	1.105	1.087	1.072	1.059	1.048

**Table 2:** Comparison of basic equations for anti-plane deformation and steady-state thermal problem.

Anti–Plains State of Deformation		Steady–State Thermal Problem	
Governing equation	$\frac{\partial^{2}W}{\partial x^{2}}+\frac{\partial^{2}W}{\partial y^{2}}=0$	Governing equation	$\frac{\partial^{2}T}{\partial x^{2}}+\frac{\partial^{2}T}{\partial y^{2}}=0$
Shear stress components	$\tau_{xz}=G\frac{\partial W}{\partial x};\;\;\tau_{yz}=G\frac{\partial W}{\partial y}$	Heat flux components	$q_{x}=-k\frac{\partial T}{\partial x};\;\;q_{y}=-k\frac{\partial T}{\partial y}$
Magnitude of the local stress	$\tau =\sqrt{\tau_{xz}^{2}+\tau_{yz}^{2}}$	Magnitude of the local heat flux	$q=\sqrt{q_{x}^{2}+q_{y}^{2}}$
Magnitude of the displacement gradient	|∇W|	Magnitude of the temperature gradient	|∇T|
Stress concentration factor *K*_t_^s^	$\frac{\tau_{\max}}{\tau_{s}}$	Analogue quantity	$\frac{q_{\max}}{q_{nom}}=\frac{{\left|\nabla T\right|}_{\max}}{{\left|\nabla T\right|}_{nom}}$
*G*—shear modulus		*k*—thermal conductivity	

**Table 3:** Comparison of *SCF*s calculated using formula (A1) (*) to the *FEM* results for a Double-V butt-welded joint subjected to tensile loading, where *θ* = 30°.

*θ* = 30°	*X* = *ρ*/(*ρ* + *L*)						
*Y* = *L*/(*L* + *t*)	0.05	0.15	0.25	0.35	0.45	0.55	0.65
0.15	2.251 2.253 *	1.717 1.720 *	1.518 1.519 *	1.400 1.400 *	1.318 1.317 *	1.255 1.255 *	1.203 1.205 *
0.25	2.257 2.254 *	1.722 1.721 *	1.521 1.519 *	1.403 1.400 *	1.321 1.317 *	1.258 1.254 *	1.205 1.204 *
0.35	2.262 2.253 *	1.724 1.720 *	1.523 1.518 *	1.403 1.398 *	1.319 1.314 *	1.254 1.250 *	1.198 1.199 *
0.45	2.250 2.243 *	1.713 1.711 *	1.511 1.508 *	1.389 1.386 *	1.303 1.301 *	1.234 1.236 *	1.175 1.183 *
0.55	2.198 2.200 *	1.670 1.673 *	1.469 1.472 *	1.347 1.351 *	1.260 1.266 *	1.191 1.201 *	1.134 1.149 *
0.65	2.084 2.079 *	1.581 1.576 *	1.389 1.384 *	1.274 1.270 *	1.193 1.192 *	1.132 1.133 *	1.085 1.086 *

**Table 4:** Comparison of SCFs calculated using formula (A2) (*) to the *FEM* results for a Double-V butt-welded joint subjected to bending load, where *θ* = 30°.

*θ* = 30°	*X* = *ρ*/(*ρ* + *L*)						
*Y* = *L*/(*L* + *t*)	0.05	0.15	0.25	0.35	0.45	0.55	0.65
0.15	2.245 2.226 *	1.712 1.699 *	1.512 1.500 *	1.394 1.381 *	1.311 1.299 *	1.247 1.236 *	1.194 1.186 *
0.25	2.218 2.214 *	1.690 1.690 *	1.491 1.490 *	1.373 1.372 *	1.291 1.289 *	1.226 1.227 *	1.172 1.176 *
0.35	2.157 2.169 *	1.643 1.654 *	1.449 1.458 *	1.333 1.341 *	1.252 1.260 *	1.189 1.198 *	1.137 1.148 *
0.45	2.057 2.068 *	1.568 1.576 *	1.384 1.390 *	1.276 1.280 *	1.201 1.204 *	1.144 1.147 *	1.099 1.102 *
0.55	1.925 1.913 *	1.472 1.462 *	1.305 1.296 *	1.209 1.201 *	1.146 1.138 *	1.101 1.093 *	1.067 1.060 *
0.65	1.772 1.780 *	1.365 1.372 *	1.223 1.227 *	1.146 1.150 *	1.099 1.101 *	1.067 1.069 *	1.044 1.048 *

**Table 5:** Comparison of SCFs calculated using formula (A3) (*) to the *FEM* results for a Double-V butt-welded joint subjected to shearing load, where *θ* = 30°.

*θ* = 30°	*X* = *ρ*/(*ρ* + *L*)						
*Y* = *L*/(*L* + *t*)	0.05	0.15	0.25	0.35	0.45	0.55	0.65
0.15	1.604 1.604 *	1.369 1.369 *	1.271 1.272 *	1.211 1.212 *	1.167 1.169 *	1.133 1.135 *	1.105 1.107 *
0.25	1.596 1.596 *	1.361 1.362 *	1.264 1.265 *	1.204 1.205 *	1.161 1.162 *	1.126 1.128 *	1.098 1.100 *
0.35	1.579 1.579 *	1.347 1.347 *	1.251 1.252 *	1.191 1.192 *	1.148 1.150 *	1.114 1.116 *	1.086 1.088 *
0.45	1.550 1.550 *	1.323 1.323 *	1.229 1.229 *	1.171 1.171 *	1.129 1.130 *	1.097 1.098 *	1.070 1.071 *
0.55	1.509 1.508 *	1.288 1.288 *	1.198 1.198 *	1.143 1.143 *	1.105 1.104 *	1.075 1.075 *	1.052 1.052 *
0.65	1.452 1.452 *	1.242 1.243 *	1.159 1.159 *	1.110 1.109 *	1.077 1.076 *	1.053 1.052 *	1.036 1.034 *

**Table 6:** Comparison of *SCF*s calculated using formula (A4) (*) to the results obtained from Equation (16) for a Single-V butt-welded joint subjected to tension, when *w*/*t* = 1.46.

*ρ*/*t*	θ*					
	10°	20°	30°	40°	50°	60°
0.01	1.79 * 1.83	2.53 * 2.56	3.19 * 3.19	3.71 * 3.75	4.12 * 4.26	4.41 * 4.73
0.025	1.64 * 1.67	2.15 * 2.19	2.55 * 2.60	2.84 * 2.91	3.03 * 3.16	3.16 * 3.36
0.05	1.53 * 1.53	1.90 * 1.92	2.16 * 2.19	2.33 * 2.37	2.43 * 2.49	2.48 * 2.57
0.1	1.43 * 1.41	1.69 * 1.68	1.84 * 1.84	1.92 * 1.94	1.96 * 1.99	1.98 * 2.00
0.2	1.34 * 1.30	1.50 * 1.48	1.58 * 1.58	1.61 * 1.62	1.62 * 1.63	1.62 * 1.62
0.4	1.26 * 1.22	1.34 * 1.34	1.37 * 1.39	1.38 * 1.41	1.38 * 1.40	1.38 * 1.38
*H*/*t*	0.06387	0.12872	0.19560	0.26570	0.34041	0.42147

## Nomenclature

*G*	shear modulus
*H*	height of the excess weld metal
*k*	thermal conductivity
*K*_t_	stress concentration factor (*SCF*)
*K*_t_^t^ *^sym^*	stress concentration factor for tensile (axial) load, Double-V weld
*K*_t_^t^ *^asym^*	stress concentration factor for tensile (axial) load, Single-V weld
*K*_t_^b^ *^sym^*	stress concentration factor for bending load, Double-V weld
*K*_t_^b^ *^asym^*	stress concentration factor for bending load, Single-V weld
*K*_t_^s^ *^sym^*	stress concentration factor for shearing load, Double-V weld
*K*_t_^s^ *^asym^*	stress concentration factor for shearing load, Single-V weld
*L*	theoretical width of the butt weld
*n*	stress field exponent for a sharp corner for tensile and bending load
*n*_s_	stress field exponent for a sharp corner for shearing load
*q*	magnitude of the heat flux
*q_max_*	magnitude of the maximum heat flux
*q_nom_*	magnitude of the nominal heat flux at the right end of the body
*R*	radius of the excess weld metal
*t*	thickness of the main plate
*t^sym^*	thickness of the main plate of a Double-V butt weld subjected to shear
*t^asym^*	thickness of the main plate of a Single-V butt weld subjected to shear
*T*	temperature
|∇*Τ*|	magnitude of the temperature gradient
|∇*Τ*|_max_	magnitude of the maximum temperature gradient
|∇*Τ*|_nom_	magnitude of the nominal temperature gradient at the right end of the body
*w*	measurable total width of the butt weld
*W*	displacement component corresponding to anti-plane deformation in z direction
*x,y,z*	Cartesian coordinates
*X = ρ/(ρ + L)*	normalized weld toe radius parameter
*Y = L/(L + t)*	normalized weld width parameter
2*α*	total angle of the sharp corner
*θ*	theoretical weld toe angle
*θ**	measurable weld toe angle
*λ*	eigenvalue of the characteristic equation corresponding to normal load
*λ*_s_	eigenvalue of the characteristic equation corresponding to shearing load
*ρ*	weld toe radius
*σ*_t_	nominal tensile (axial) stress
*σ*_b_	nominal bending stress
*σ*_1max_	maximum principal stress at the weld toe due to tensile or bending load
*τ*	shearing stress
*τ*_s_	nominal shearing stress corresponding to anti-plane deformation
*τ_max_*	maximum shear stress at the weld toe due to shear stress longitudinal to the weld
*Ψ*	potential function
*∂Ψ/∂n*	partial derivative normal to the bonding contour

## Appendix A

Formulas for calculating *SCF*s for a Double-V and a Single-V butt-welded joint under tension, bending and shear.

Double-V butt-welded joint–tensile load

(A1) $$K_{t}^{t\;sym}=X^{n}\left(A_{0}^{t}+A_{1}^{t}X+A_{2}^{t}X^{1.1}\right)$$

where: range of application: 0<X≤2/3; 0≤Y≤2/3; 0≤θ≤π/2

$$A_{0}^{t}=A_{00}^{t}+A_{01}^{t}Y^{5}+A_{02}^{t}Y^{6}$$

$$A_{00}^{t}=1+1.703\theta^{0.75}-1.591\theta -0.860\theta^{2}+0.709\theta^{3}-0.153\theta^{4}$$

$$A_{01}^{t}=-1.672\theta +9.310\theta^{2}-8.407\theta^{3}+2.216\theta^{4}$$

$$A_{02}^{t}=-2.768\theta -5.558\theta^{2}+8.088\theta^{3}-2.499\theta^{4}$$

$$A_{1}^{t}=A_{10}^{t}+A_{11}^{t}Y^{5.25}+A_{12}^{t}Y^{5.5}+A_{13}^{t}Y^{6}$$

$$A_{10}^{t}=1.510\theta^{0.75}-21.753\theta^{2}+60.095\theta^{3}-59.047\theta^{4}+26.468\theta^{5}-4.594\theta^{6}$$

$$A_{11}^{t}=3347.164\theta^{2.5}-484.574\theta^{4}$$

$$A_{12}^{t}=-5482.603\theta^{2.5}+732.855\theta^{4}$$

$$A_{13}^{t}=2150.066\theta^{2.5}-231.877\theta^{4}$$

$$A_{2}^{t}=A_{20}^{t}+A_{21}^{t}Y^{5.25}+A_{22}^{t}Y^{5.5}+A_{23}^{t}Y^{6}$$

$$A_{20}^{t}=-2.129\theta^{0.75}+23.927\theta^{2}-61.415\theta^{3}+58.649\theta^{4}-25.829\theta^{5}+4.425\theta^{6}$$

$$A_{21}^{t}=-3505.908\theta^{2.5}+484.581\theta^{4}$$

$$A_{22}^{t}=5691.409\theta^{2.5}-710.326\theta^{4}$$

$$A_{23}^{t}=-2192.311\theta^{2.5}+204.792\theta^{4}$$

Double-V butt-welded joint–bending load

(A2) $$K_{t}^{b\;sym}=X^{n}\left(A_{0}^{b}+A_{1}^{b}X+A_{2}^{b}X^{1.1}\right)$$

where: range of application: 0<X≤2/3; 0≤Y≤2/3; 0≤θ≤π/2

$$A_{0}^{b}=A_{00}^{b}+A_{01}^{b}Y^{5}+A_{02}^{b}Y^{6}$$

$$A_{00}^{b}=1+1.484\theta^{0.75}-1.334\theta -0.926\theta^{2}+0.756\theta^{3}-0.170\theta^{4}$$

$$A_{01}^{b}=-30.294\theta +55.779\theta^{2.5}-56.321\theta^{3.5}+23.951\theta^{4}$$

$$A_{02}^{b}=38.179\theta -73.520\theta^{2.5}+75.297\theta^{3.5}-32.206\theta^{4}$$

$$A_{1}^{b}=A_{10}^{b}+A_{11}^{b}Y^{5}+A_{12}^{b}Y^{6}+A_{13}^{b}Y^{7}$$

$$A_{10}^{b}=2.312\theta -21.029\theta^{2}+54.420\theta^{3}-52.168\theta^{4}+23.152\theta^{5}-4.022\theta^{6}$$

$$A_{11}^{b}=-314.24\theta +3564.88\theta^{2}-8769.47\theta^{3}+8002.34\theta^{4}-3330.18\theta^{5}+533.64\theta^{6}$$

$$A_{12}^{b}=1025.2\theta -11913.7\theta^{2}+28711.0\theta^{3}-26287.0\theta^{4}+11020.5\theta^{5}-1779.3\theta^{6}$$

$$A_{13}^{b}=-859.1\theta +9788.0\theta^{2}-22921.6\theta^{3}+20762.3\theta^{4}-8633.3\theta^{5}+1382.0\theta^{6}$$

$$A_{2}^{b}=A_{2}^{b}+A_{2}^{b}Y^{5}+A_{2}^{b}Y^{6}+A_{2}^{b}Y^{7}$$

$$A_{20}^{b}=-3.593\theta +25.590\theta^{2}-60.017\theta^{3}+55.916\theta^{4}-24.518\theta^{5}+4.233\theta^{6}$$

$$A_{21}^{b}=363.50\theta -3822.05\theta^{2}+8964.60\theta^{3}-8059.43\theta^{4}+3330.82\theta^{5}-531.86\theta^{6}$$

$$A_{22}^{b}=-1183.2\theta +12837.1\theta^{2}-29545.4\theta^{3}+26651.4\theta^{4}-11096.5\theta^{5}+1785.4\theta^{6}$$

$$A_{23}^{b}=987.1\theta -10552.8\theta^{2}+23619.1\theta^{3}-21060.0\theta^{4}+8688.1\theta^{5}-1384.5\theta^{6}$$

Double-V butt-welded joint–shearing load

(A3) $$K_{t}^{s\;sym}=X^{n_{s}}\left(A_{0}^{s}+A_{1}^{s}X+A_{2}^{s}X^{2}+A_{3}^{s}X^{3}\right).$$

where: $Y^{sym}=L/\left(L+t^{sym}\right)$, range of application: 0<X≤2/3; $0\leq Y^{sym}\leq 2/3$; 0≤θ≤π/2

$$A_{0}^{s}=A_{00}^{s}+A_{01}^{s}{\left(Y^{sym}\right)}^{3}+A_{02}^{s}{\left(Y^{sym}\right)}^{4}$$

$$A_{00}^{s}=1+0.4068\theta^{0.75}-1.2554\theta^{2}+1.3008\theta^{3}-0.6596\theta^{4}+0.1331\theta^{5}$$

$$A_{01}^{s}=-1.2337\theta +0.6550\theta^{2}-0.1106\theta^{3}$$

$$A_{02}^{s}=0.4757\theta -0.3672\theta^{2}+0.1963\theta^{3}-0.0528\theta^{4}$$

$$A_{1}^{s}=A_{10}^{s}+A_{11}^{s}{\left(Y^{sym}\right)}^{2}+A_{12}^{s}{\left(Y^{sym}\right)}^{3}+A_{13}^{s}{\left(Y^{sym}\right)}^{4}+A_{14}^{s}{\left(Y^{sym}\right)}^{5}$$

$$A_{10}^{s}=-0.3474\theta +0.5466\theta^{2}+0.0682\theta^{3}-0.0682\theta^{4}$$

$$A_{11}^{s}=-0.3487\theta^{2}+0.0671\theta^{3}$$

$$A_{12}^{s}=-1.4274\theta +3.6342\theta^{2}+1.4638\theta^{3}-3.1567\theta^{4}+0.9430\theta^{5}$$

$$A_{13}^{s}=5.7757\theta -14.9509\theta^{2}+6.2601\theta^{3}-0.5036\theta^{4}$$

$$A_{14}^{s}=-4.9147\theta +12.0219\theta^{2}-3.8935\theta^{3}$$

$$A_{2}^{s}=A_{20}^{s}+A_{21}^{s}{\left(Y^{sym}\right)}^{4}+A_{22}^{s}{\left(Y^{sym}\right)}^{5}+A_{23}^{s}{\left(Y^{sym}\right)}^{6}$$

$$A_{20}^{s}=-0.4520\theta +2.5654\theta^{2}-3.6200\theta^{3}+1.8544\theta^{4}-0.3575\theta^{5}$$

$$A_{21}^{s}=23.291\theta -53.144\theta^{2}+29.009\theta^{3}-5.837\theta^{4}$$

$$A_{22}^{s}=-77.789\theta +168.428\theta^{2}-93.726\theta^{3}+21.225\theta^{4}$$

$$A_{23}^{s}=65.663\theta -131.421\theta^{2}+71.156\theta^{3}-16.786\theta^{4}$$

$$A_{3}^{s}=A_{30}^{s}+A_{31}^{s}{\left(Y^{sym}\right)}^{3}+A_{32}^{s}{\left(Y^{sym}\right)}^{4}+A_{33}^{s}{\left(Y^{sym}\right)}^{5}+A_{34}^{s}{\left(Y^{sym}\right)}^{6}$$

$$A_{30}^{s}=0.1683\theta^{2}-2.0633\theta^{3}+3.4158\theta^{4}-2.0331\theta^{5}+0.4279\theta^{6}$$

$$A_{31}^{s}=2.7387\theta -11.7784\theta^{2}+4.0711\theta^{3}-0.4858\theta^{4}$$

$$A_{32}^{s}=-32.282\theta +83.466\theta^{2}-19.037\theta^{3}$$

$$A_{33}^{s}=86.427\theta -176.451\theta^{2}+35.193\theta^{3}$$

$$A_{34}^{s}=-65.409\theta +112.572\theta^{2}-18.945\theta^{3}$$

Single-V butt-welded joint–tensile load

(A4) $$K_{t}^{t\;asym}=X^{n}\left(A_{0}^{t}+A_{1}^{t}X+A_{2}^{t}X^{1.1}\right)$$

where: range of application: 0<X≤2/3; 0≤Y≤2/3; 0≤θ≤π/2

$$A_{0}^{t}=A_{00}^{t}+A_{01}^{t}Y^{3}+A_{02}^{t}Y^{4}$$

$$A_{00}^{t}=1+1.1074\theta^{0.75}-0.5271\theta -2.1097\theta^{2}+2.0446\theta^{3}-0.8531\theta^{4}+0.1407\theta^{5}$$

$$A_{01}^{t}=-2.4005\theta +2.0601\theta^{2}+0.6306\theta^{3}-1.1627\theta^{4}+0.3199\theta^{5}$$

$$A_{02}^{t}=0.9584\theta -3.7036\theta^{2}+6.0216\theta^{3}-6.0896\theta^{4}+3.2921\theta^{5}-0.6987\theta^{6}$$

$$A_{1}^{t}=A_{10}^{t}+A_{11}^{t}Y^{5}+A_{12}^{t}Y^{5.25}+A_{13}^{t}Y^{5.5}$$

$$A_{10}^{t}=0.9070\theta^{0.5}-19.5924\theta^{2}+60.6784\theta^{3}-63.9954\theta^{4}+30.2690\theta^{5}-5.4716\theta^{6}$$

$$A_{11}^{t}=7477.38\theta^{2}-5846.23\theta^{3}+1285.50\theta^{4}$$

$$A_{12}^{t}=-16743.10\theta^{2}+12540.39\theta^{3}-2653.18\theta^{4}$$

$$A_{13}^{t}=9357.28\theta^{2}-6701.62\theta^{3}+1354.26\theta^{4}$$

$$A_{2}^{t}=A_{20}^{t}+A_{21}^{t}Y^{5}+A_{22}^{t}Y^{5.25}+A_{23}^{t}Y^{5.5}$$

$$A_{20}^{t}=-1.2239\theta^{0.5}+20.1999\theta^{2}-58.7856\theta^{3}+60.3360\theta^{4}-28.0141\theta^{5}+4.9914\theta^{6}$$

$$A_{21}^{t}=-7866.99\theta^{2}+5610.80\theta^{3}-1146.49\theta^{4}$$

$$A_{22}^{t}=17594.2\theta^{2}-11995.8\theta^{3}+2338.7\theta^{4}$$

$$A_{23}^{t}=-9813.03\theta^{2}+6375.03\theta^{3}-1172.33\theta^{4}$$

Single-V butt-welded joint–bending load

(A5) $$K_{t}^{b\;asym}=X^{n}\left(A_{0}^{b}+A_{1}^{b}X+A_{2}^{b}X^{1.1}\right)$$

where: range of application: 0<X≤2/3; 0≤Y≤2/3; 0≤θ≤π/2

$$A_{0}^{b}=A_{00}^{b}+A_{01}^{b}Y^{3}+A_{02}^{b}Y^{4}$$

$$A_{00}^{b}=1+1.1795\theta^{0.75}-0.6774\theta -1.8094\theta^{2}+1.7034\theta^{3}-0.6927\theta^{4}+0.1135\theta^{5}$$

$$A_{01}^{b}=-1.8984\theta +1.0254\theta^{2}+9.9490\theta^{3}-14.8128\theta^{4}+8.0715\theta^{5}-1.5835\theta^{6}$$

$$A_{02}^{b}=-0.1129\theta^{2}-10.6173\theta^{3}+16.1861\theta^{4}-9.0484\theta^{5}+1.8049\theta^{6}$$

$$A_{1}^{b}=A_{10}^{b}+A_{11}^{b}Y^{5}+A_{12}^{b}Y^{5.25}+A_{13}^{b}Y^{5.5}$$

$$A_{10}^{b}=8.6792\theta^{0.75}-14.9200\theta +7.1263\theta^{2}+8.2688\theta^{3}-8.7331\theta^{4}+2.1718\theta^{5}$$

$$A_{11}^{b}=6924.89\theta^{2}-9279.74\theta^{3}-2437.21\theta^{4}+8431.29\theta^{5}-2910.74\theta^{6}$$

$$A_{12}^{b}=-14637.24\theta^{2}+18486.92\theta^{3}+6589.89\theta^{4}-18798.04\theta^{5}+6396.51\theta^{6}$$

$$A_{13}^{b}=7683.85\theta^{2}-9027.16\theta^{3}-4421.30\theta^{4}+10556.38\theta^{5}-3532.62\theta^{6}$$

$$A_{2}^{b}=A_{20}^{b}+A_{21}^{b}Y^{5}+A_{22}^{b}Y^{5.25}+A_{23}^{b}Y^{5.5}$$

$$A_{20}^{b}=-0.9558\theta^{0.5}+16.6843\theta^{2}-45.4073\theta^{3}+42.5769\theta^{4}-18.2659\theta^{5}+3.0640\theta^{6}$$

$$A_{21}^{b}=-755.879\theta^{2}-2293.872\theta^{3}+2749.303\theta^{4}-1090.224\theta^{5}$$

$$A_{22}^{b}=1.875\theta^{2}+9543.03\theta^{3}-9328.61\theta^{4}+3200.30\theta^{5}$$

$$A_{23}^{b}=962.36\theta^{2}-7792.15\theta^{3}+6987.82\theta^{4}-2216.02\theta^{5}$$

Single-V butt-welded joint–shearing load

(A6) $$K_{t}^{s\;asym}=X^{n_{s}}\left(A_{0}^{s}+A_{1}^{s}X+A_{2}^{s}X^{2}+A_{3}^{s}X^{3}\right)$$

where: $Y^{asym}=L/\left(L+t^{asym}\right)$, range of application: 0<X≤2/3; $0\leq Y^{asym}\leq 4/5$; 0≤θ≤π/2

$$A_{0}^{s}=A_{00}^{s}+A_{01}^{s}{\left(\frac{Y^{asym}}{2-Y^{asym}\;}\right)}^{3}+A_{02}^{s}{\left(\frac{Y^{asym}}{2-Y^{asym}\;}\right)}^{4}$$

$$A_{1}^{s}=A_{10}^{s}+A_{11}^{s}{\left(\frac{Y^{asym}}{2-Y^{asym}\;}\right)}^{2}+A_{12}^{s}{\left(\frac{Y^{asym}}{2-Y^{asym}\;}\right)}^{3}+A_{13}^{s}{\left(\frac{Y^{asym}}{2-Y^{asym}\;}\right)}^{4}+A_{14}^{s}{\left(\frac{Y^{asym}}{2-Y^{asym}\;}\right)}^{5}$$

$$A_{2}^{s}=A_{20}^{s}+A_{21}^{s}{\left(\frac{Y^{asym}}{2-Y^{asym}}\right)}^{4}+A_{22}^{s}{\left(\frac{Y^{asym}}{2-Y^{asym}\;}\right)}^{5}+A_{23}^{s}{\left(\frac{Y^{asym}}{2-Y^{asym}\;}\right)}^{6}$$

$$A_{3}^{s}=A_{30}^{s}+A_{31}^{s}{\left(\frac{Y^{asym}}{2-Y^{asym}\;}\right)}^{3}+A_{32}^{s}{\left(\frac{Y^{asym}}{2-Y^{asym}\;}\right)}^{4}+A_{33}^{s}{\left(\frac{Y^{asym}}{2-Y^{asym}\;}\right)}^{5}+A_{34}^{s}{\left(\frac{Y^{asym}}{2-Y^{asym}\;}\right)}^{6}$$

Formulas for calculating $A_{ij}^{s}$ coefficients are the same as for a Double-V butt-welded joint that has been subjected to shear.
